# Supplementary figures and images for: Interferon-gamma promotes iron export in human macrophages to limit intracellular bacterial replication
Source: PLoS One. 2020 Dec 8;15(12):e0240949. doi: 10.1371/journal.pone.0240949 (PMC7723272; doi:10.1371/journal.pone.0240949)

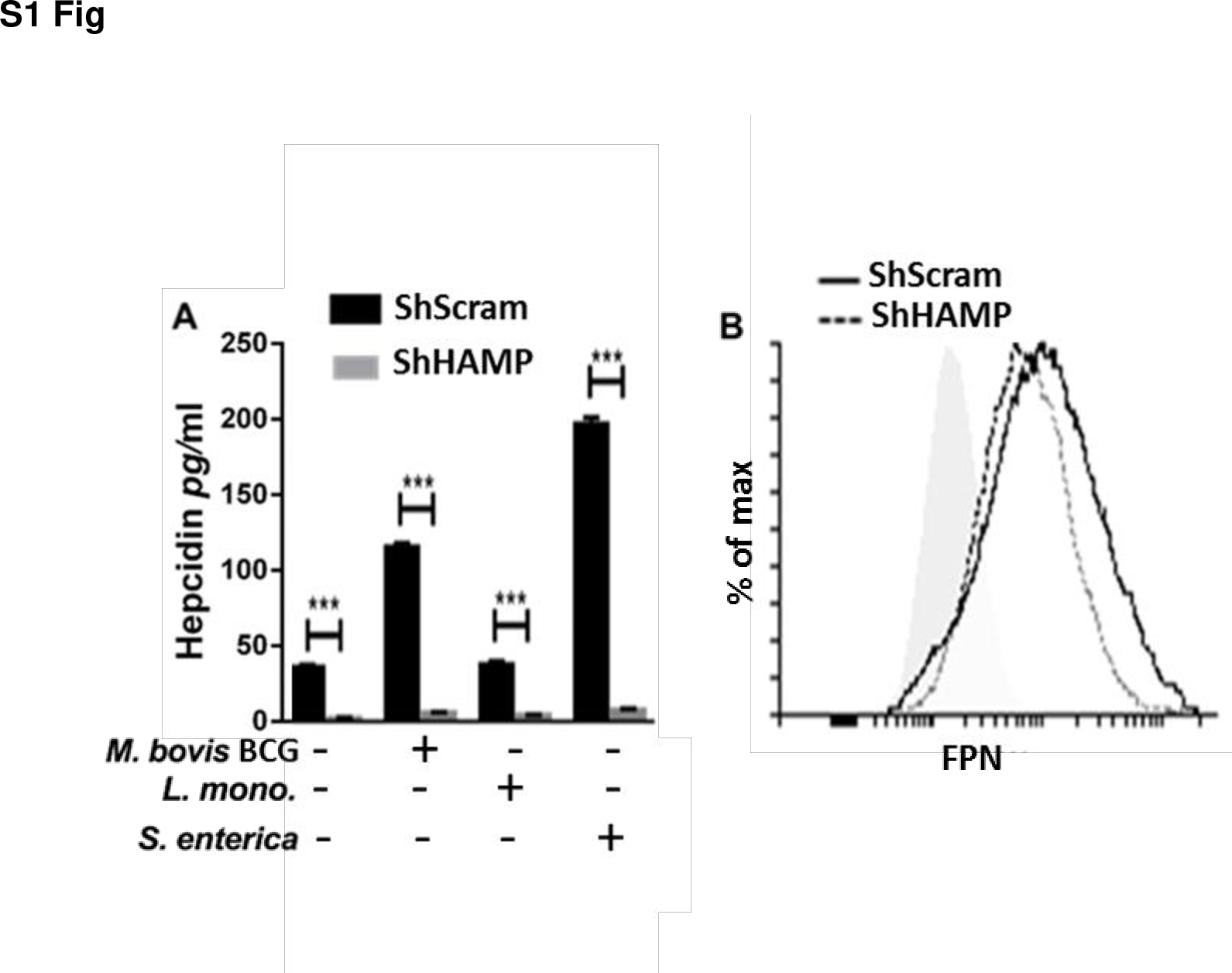

Supplement: S1 Fig — A) Hepcidin secretion in ShHAMP THP-1 macrophages and respective ShScram controls after infection with M. bovis BCG, L. monocytogenes and S. enterica. B) Surface ferroportin expression in ShHAMP THP-1 macrophages and respective ShScram controls measured by flow cytometry. (TIF) [file pone.0240949.s001.tif]

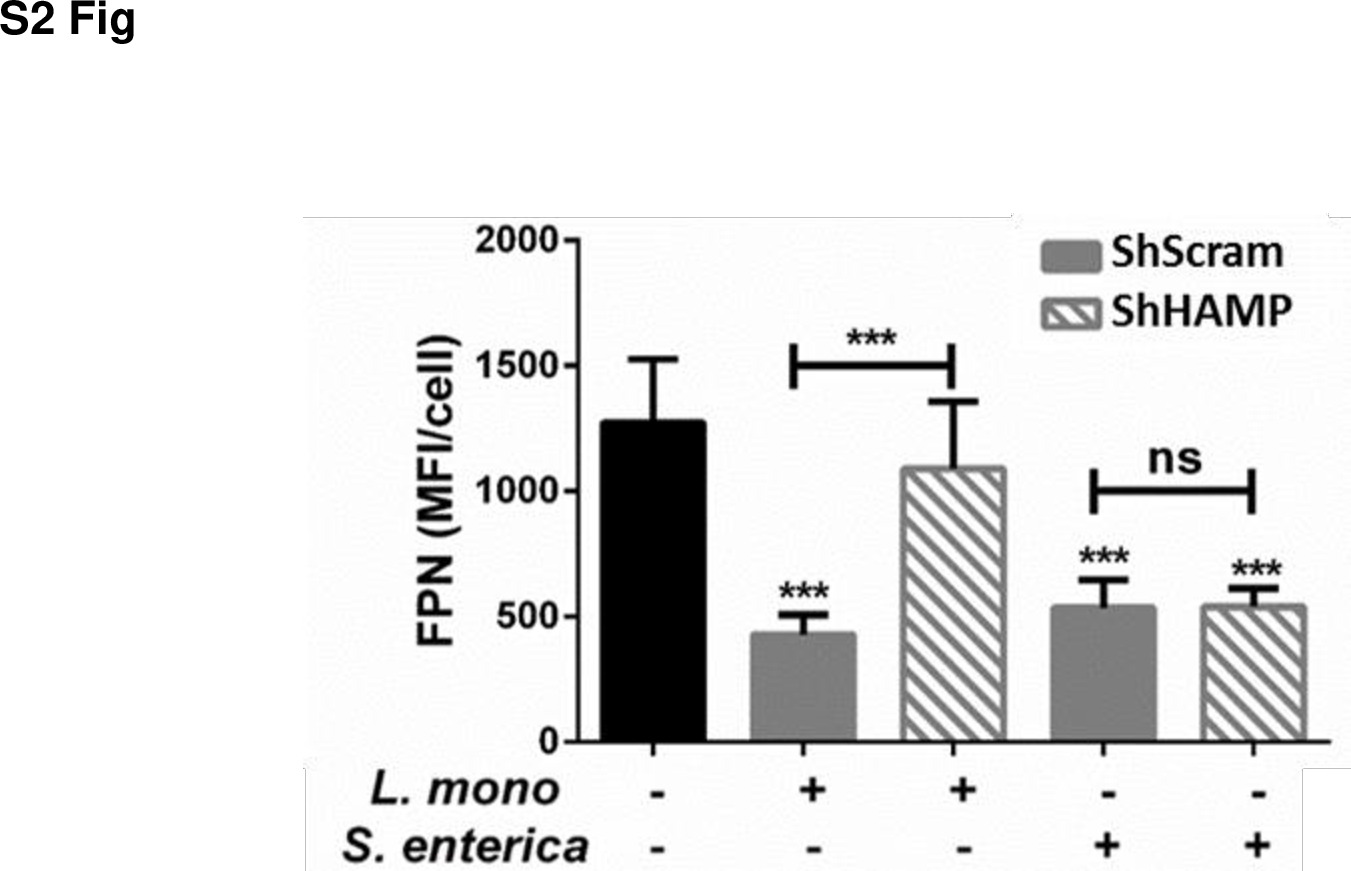

Supplement: S2 Fig — Ferroportin expression in ShHAMP THP-1 macrophages eight hours post-infection with L. monocytogenes and 16 hours post-infection with S. enterica. Ferroportin levels were quantified by mean fluorescence intensity of 40 cells from three different fields of three independent experiments. ***p<0.001. (TIF) [file pone.0240949.s002.tif]

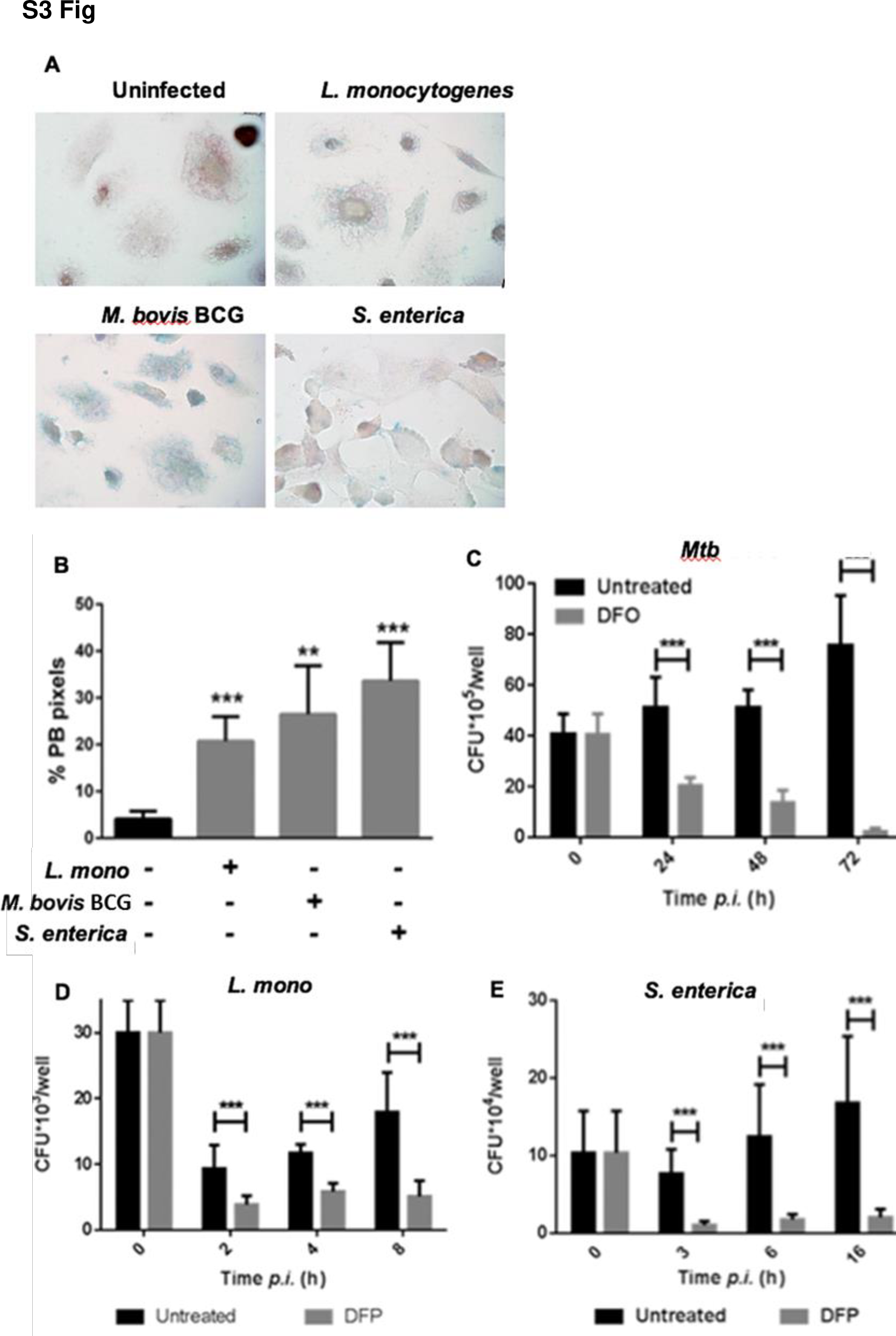

Supplement: S3 Fig — A) Intracellular iron Prussian Blue staining in macrophages infected with three siderophilic bacteria. B) Percentage of Prussian Blue (PB) pixels in THP-1 macrophages after infection with three siderophilic bacteria. (C) Mycobacterium tuberculosis intracellular burden in THP-1 macrophages in presence of iron chelator DFO. D) Listeria monocytogenes intracellular burden in THP-1 macrophages in presence of iron chelator DFP. E) Salmonella enterica intracellular burden in THP-1 macrophages in presence of iron chelator DFP. **p<0.01, ***p<0.001. All data were from three independent experiments. (TIF) [file pone.0240949.s003.tif]

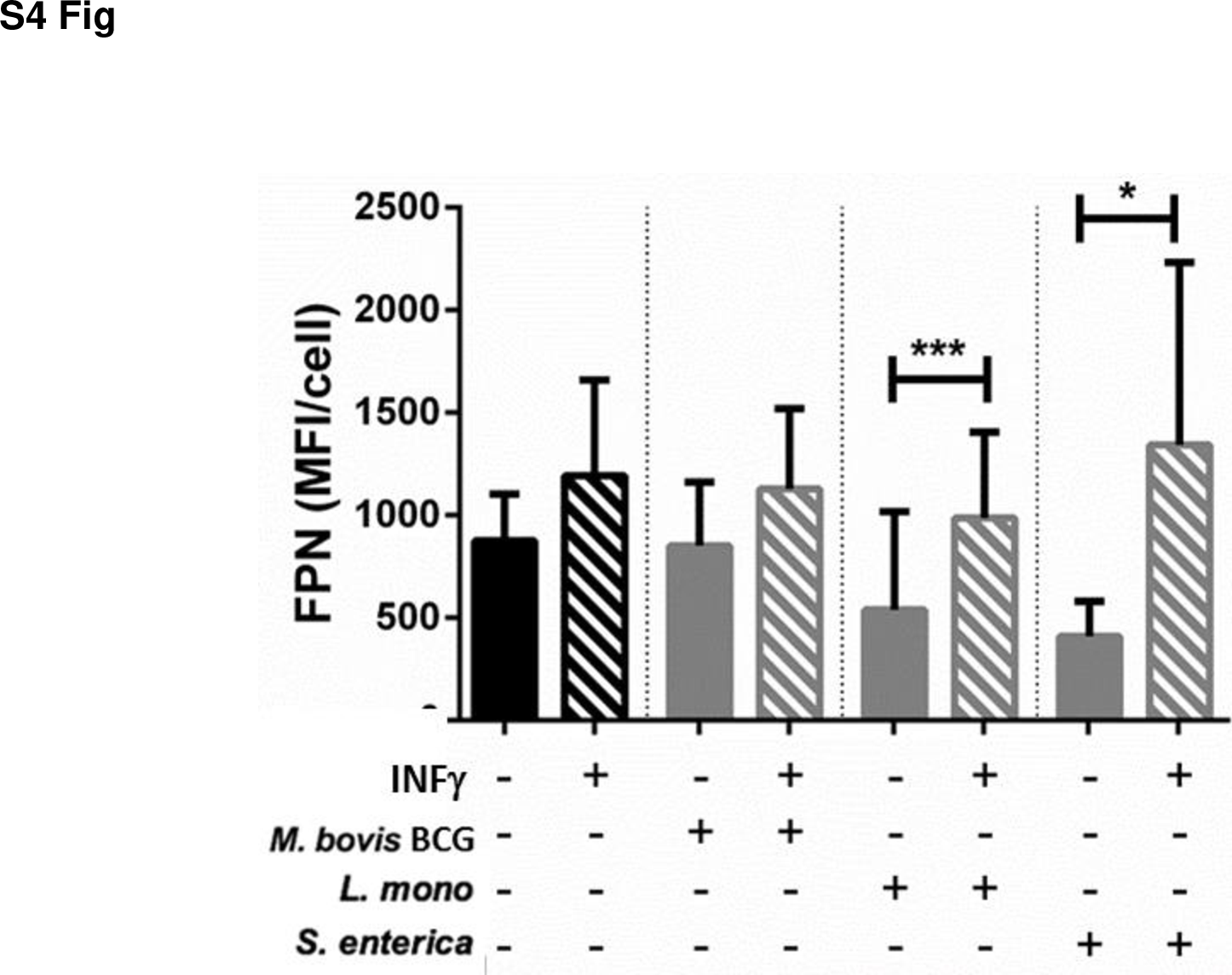

Supplement: S4 Fig — Ferroportin in IFN-γ-treated THP-1 macrophages eight hours post-infection with L. monoytogenes, 16 hours post-infection with S. enterica and 24 hours post-infection with M. bovis BCG bacteria. Ferroportin levels were quantified by mean fluorescence intensity of 40 cells from three different fields of three independent experiments. *p<0.05, ***p<0.001. (TIF) [file pone.0240949.s004.tif]

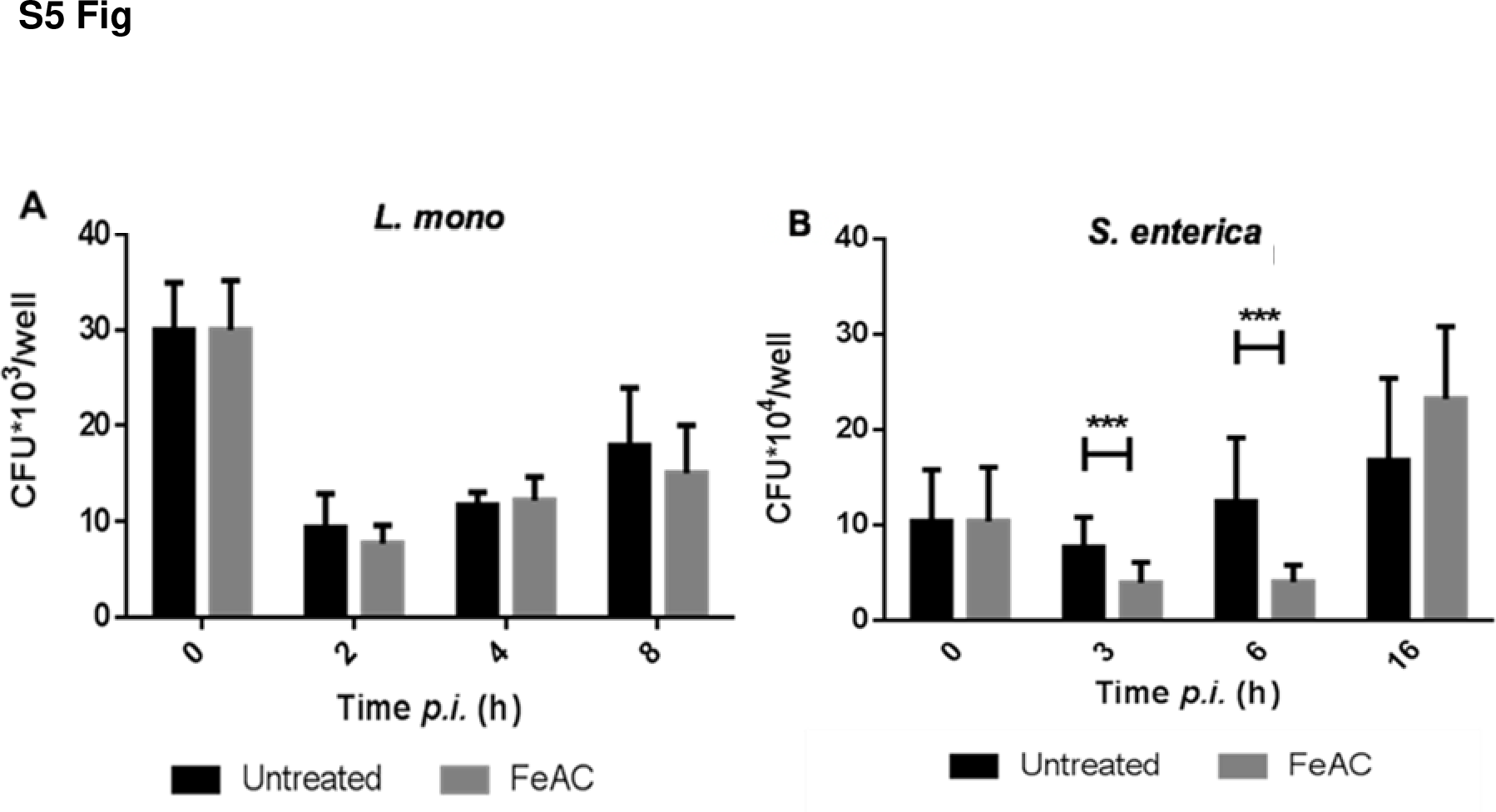

Supplement: S5 Fig — A) THP-1 macrophages differentiated as described in Materials and Methods, rested and infected in iron-supplemented medium. Listeria monocytogenes (A) and S. enterica (B) intracellular bacterial burdens were determined by a gentamicin protection assay. ***p<0.001. Data were from three independent experiments. (TIF) [file pone.0240949.s005.tif]

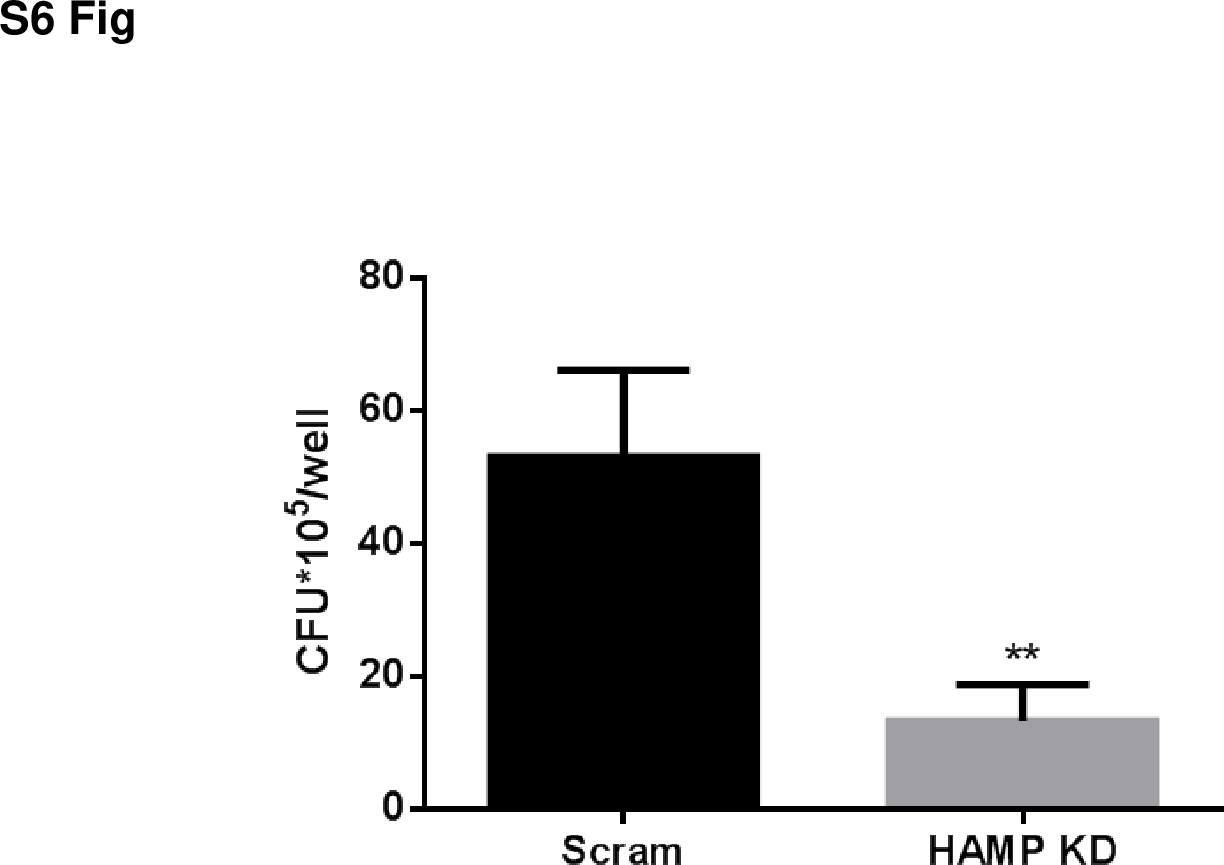

Supplement: S6 Fig — Mycobacterium bovis BCG intracellular burden in ShHAMP THP-1 macrophages 24 hours post-infection. **p<0.01. Data were from three independent experiments. (TIF) [file pone.0240949.s006.tif]
